# The Editor's Letter-Box

**Published:** 1887-11-26

**Authors:** Mary Wardell

**Affiliations:** 55, Stanley Gardens, Belsize, N.W.


In this month's number of The Hospital I observe a paper on "Scarlet Fever—the Dangers of Convalescence." The writer urges the establishment of Scarlet Fever Convalescent Homes, and seems to be unaware that this need was first brought under public notice by me more than eight years ago, and after five years of effort, the first convalescent home for any infectious disease was opened by the Prince and Princess of Wales, at Stanmore, within ten miles of London by road. During the three years of its existence more than 550 persons have availed themselves of its benefits, a small number of whom came as first-class patients, and paid sufficient to cover their cost; but by far the larger number were women and children of the lower middle and working classes, who are admitted at charges far below the expenses of maintaining them, one of the heavy expenses being the keep of an omnibus, horse and man, for fetching the patients from door to door, and so avoiding all risk to the public by their removal to fresh air and exercise in a large garden. Unhappily, the donations have not been on a sufficiently generous scale to enable the whole plan to be carried out, and as the Home is burdened with debt, and the subscriptions are inadequate to meet the current expenditure, the donations cannot be put aside to accumulate for a building fund for completing the whole plan. I enclose papers respecting my Home, and will be happy to send them and give any further information to the writer of the article or to anyone who may wish to know more of the institution.

(Miss) Mary Wardell.

55, Stanley Gardens, Belsize, N.W., Nov. 17th, 1887.

